# Identification and genotyping of *Enterocytozoon bieneusi* in wild Himalayan marmots (*Marmota himalayana*) and Alashan ground squirrels (*Spermophilus alashanicus*) in the Qinghai-Tibetan Plateau area (QTPA) of Gansu Province, China

**DOI:** 10.1186/s13071-020-04233-9

**Published:** 2020-07-22

**Authors:** Jie Xu, Xin Wang, Huaiqi Jing, Shengkui Cao, Xiaofan Zhang, Yanyan Jiang, Jianhai Yin, Jianping Cao, Yujuan Shen

**Affiliations:** 1 National Institute of Parasitic Diseases, Chinese Center for Disease Control and Prevention, Shanghai, 200025 China; 2 Chinese Center for Tropical Diseases Research, Shanghai, 200025 China; 3 WHO Collaborating Centre for Tropical Diseases, Shanghai, 200025 China; 4 National Center for International Research on Tropical Diseases, Ministry of Science and Technology, Shanghai, 200025 China; 5 Key Laboratory of Parasite and Vector Biology, Ministry of Health, Shanghai, 200025 China; 6 National Institute of Infectious Diseases, Chinese Center for Disease Control and Prevention, Beijing, 102206 China

**Keywords:** *Enterocytozoon bieneusi*, Himalayan marmots, Alashan ground squirrels, Prevalence, Qinghai-Tibetan Plateau area

## Abstract

**Background:**

*Enterocytozoon bieneusi* is the most frequently detected microsporidian species in humans and animals. Currently, to the best of our knowledge, no information on *E. bieneusi* infection in Himalayan marmots (*Marmota himalayana*) and Alashan ground squirrels (*Spermophilus alashanicus*) is available worldwide. The aim of the present study was to understand the occurrence and genetic characterizations of *E. bieneusi* in Himalayan marmots and Alashan ground squirrels in the Qinghai-Tibetan Plateau area (QTPA), Gansu Province, China.

**Methods:**

A total of 498 intestinal contents were collected from 399 Himalayan marmots and 99 Alashan ground squirrels in QTPA. These samples were screened for the presence of *E. bieneusi* by using nested polymerase chain reaction and sequencing of the internal transcribed spacer (ITS) region of the ribosomal RNA (rRNA) gene. The ITS-positive sequences were aligned and phylogenetically analyzed to determine the genotypes of *E. bieneusi*.

**Results:**

The average infection rate of *E. bieneusi* was 10.0% (50/498), with 11.8% (47/399) in Himalayan marmots and 3.0% (3/99) in Alashan ground squirrels. A total of 7 distinct *E. bieneusi* genotypes were confirmed: 1 known genotype, YAK1 (*n* = 18) and 6 novel genotypes, named as ZY37 (*n* = 27), HN39 (*n* = 1), HN96 (*n* = 1), SN45 (*n* = 1), XH47 (*n* = 1) and ZY83 (*n* = 1). All the genotypes obtained in the present study were classified into group 1.

**Conclusions:**

To our knowledge, this is the first report of *E. bieneusi* in Himalayan marmots and Alashan ground squirrels in China. The identification of genotype YAK1 in the two rodent species expanded the host range of this genotype. All the seven genotypes were clustered into zoonotic group 1, suggesting that these animal species can be potential epidemiological vectors of zoonotic microsporidiosis caused by *E. bieneusi* and pose a threat to ecological security. It is necessary to strengthen management practices and surveillance in the investigated areas to reduce the risk of *E. bieneusi* infection from the two rodent species to humans.
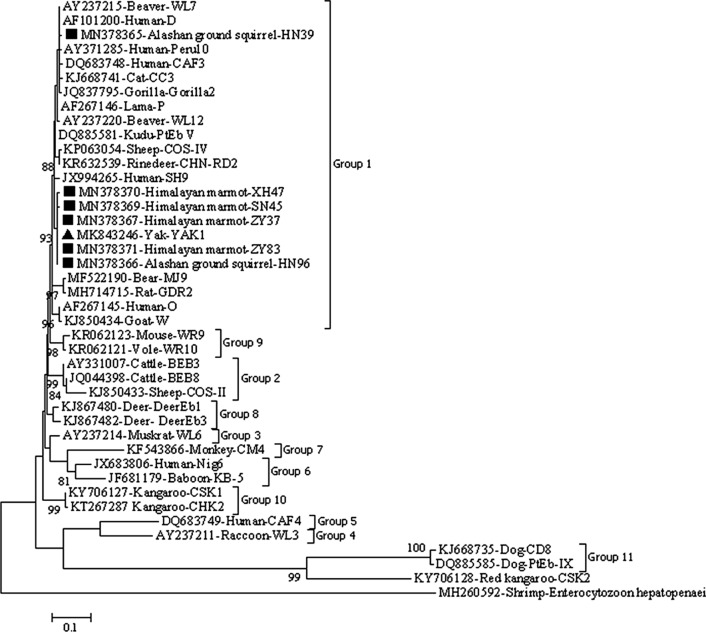

## Background

Microsporidia are a large phylum of fungus-related pathogens with an obligate intracellular lifestyle, which are composed of at least 1500 species within 200 genera, and 17 of these species have been reported to cause human infections [1, 2]. *Enterocytozoon bieneusi* is the most frequently detected species (more than 90%) in microsporidiosis cases [3]. Microsporidiosis caused by *E. bieneusi* is characterized by asymptomatic infection or self-limiting diarrhea in immunocompetent and healthy humans, and life-threatening and persistent diarrhea in immunocompromised individuals, such as HIV-positive patients, cancer patients, organ transplant recipients, and children [3]. *Enterocytozoon bieneusi* has also been widely reported in numerous mammalian and avian hosts [4]. In fact, wild animals can play a role in spreading microsporidium spores to humans and farmlands [5]. Consumption of *E. bieneusi* spores from human and animal feces *via* contaminated food or water is the most probable transmission route of *E. bieneusi* to humans or animals [3]. *Enterocytozoon bieneusi* has been detected in foods such as milk, raspberries, cucumbers, beans and lettuce, which can act as intermediate carriers of this parasite [6–8]. In 2009, a food-borne microsporidiosis outbreak caused by *E. bieneusi* was reported in Sweden [7]. No water-borne microsporidiosis outbreak has been reported to date; however, *E. bieneusi* spores have been found in multiple water bodies, for example drinking-source watersheds [9], recreational watersheds [10], and wastewater resources used for irrigation of farmlands [11], indicating the possibility of water-borne transmission.

Significant progress has been made in understanding the host specificity and transmission patterns of *E. bieneusi* by using genotyping tools and phylogenetic analyses. Currently, the standard method for identifying and genotyping *E. bieneusi* isolates is the analysis of genetic polymorphisms in the ribosomal internal transcribed spacer (ITS) region [12]. To date, almost 500 genotypes have been identified in a wide range of host species and divided into 11 different genetic groups [13]. At least 106 genotypes have been found in humans, with 46 of them detected in animals [14]. Groups 1 and 2 are zoonotic in nature and mostly composed of genotypes that spread in human and animal hosts [15]. To better understand the epidemiology of *E. bieneusi* and assess the role of animals in its transmission to humans, molecular epidemiological studies should be conducted for genotyping *E. bieneusi* isolates from under-sampled animal hosts.

Rodents are well-known hosts or reservoirs for a number of zoonotic infectious diseases (e.g. plague, leptospirosis, leishmaniasis, salmonellosis and viral hemorrhagic fevers) and may act as possible mediators of these diseases [16]. Previous epidemiological studies of *E. bieneusi* in rodents have identified 56 valid genotypes [9, 17–28], with 31 and 8 genotypes belonging to group 1 and 2, respectively (Additional file 1: Table S1). However, there is limited genetic information on *E. bieneusi* in rodent species belonging to genera *Marmota* and *Spermophilus*. To date, only one study has reported the occurrence of *E. bieneusi* in a species of the genus *Marmota*, i.e. wild woodchucks (*Marmota monax*) [9]. In China, Himalayan marmots (*Marmota himalayana*) and Alashan ground squirrels (*Spermophilus alashanicus*) are common wild rodents in plateau areas, and they often share the same pasture with humans, herbivorous animals, and other wild animals. However, to the best of our knowledge, no reports are available on the occurrence and genetic characterizations of *E. bieneusi* in the two rodent species in these areas. The aims of the present study were to determine the epidemiological status and genotypes of *E. bieneusi* in the two wild animals in the Qinghai-Tibetan Plateau Area (QTPA) in China by nested polymerase chain reaction (PCR) and sequence analysis of the ITS region of the ribosomal RNA (rRNA) gene, and assess the potential zoonotic transmission.

## Methods

### Sample collection

A total of 498 intestinal contents were collected from 399 Himalayan marmots and 99 Alashan ground squirrels from June to September 2017 in QTPA in Gansu Province (geographical coordinates: 32°31′–42°57′N latitude, 92°13′–108°46′E longitude), China. The Himalayan marmots were obtained from 4 cities/counties, namely Luqu (*n* = 98), Sunan (*n* = 100), Xiahe (*n* = 102) and Zhangye (*n* = 99), whereas the Alashan ground squirrels were from Huining County (*n* = 99) (Table 1). The animals were captured alive with mousetraps in the field, and the intestinal contents were collected after euthanizing the animals with a high-dose of CO_2_ in the laboratory of the local Center for Disease Control and Prevention (CDC) with security measures. All the collected intestinal content samples were stored in 2 ml sterile tubes at − 20 °C, and then transported in a cooler with ice packs to our laboratory prior to DNA extraction in Shanghai.

**Table 1:** Prevalence and distribution of *E. bieneusi* genotypes in wild Himalayan marmots and Alashan ground squirrels

Host	Collection site	No. examined	No. positive (%)	Genotype
Himalayan marmot (*Marmota himalayana*)	Luqu	98	9 (9.2)	YAK1 (*n* = 9)
	Sunan	100	13 (13.0)	ZY37 (*n* = 12), SN45 (*n* = 1)
	Xiahe	102	8 (7.8)	YAK1 (*n* = 7), XH47 (*n* = 1)
	Zhangye	99	17 (17.2)	ZY37 (*n* = 15), YAK1 (*n* = 1), ZY83 (*n* = 1)
	Subtotal	399	47 (11.8)	ZY37 (*n* = 27), YAK1 (*n* = 17), SN45 (*n* = 1), XH47 (*n* = 1), ZY83 (*n* = 1)
Alashan ground squirrel (*Spermophilus alaschanicus*)	Huining	99	3 (3.0)	HN39 (*n* = 1), HN96 (*n* = 1), YAK1 (*n* = 1)
Total		498	50 (10.0)	ZY37 (*n* = 27), YAK1 (*n* = 18), HN39 (*n* = 1), HN96 (*n* = 1), SN45 (*n* = 1), XH47 (*n* = 1), ZY83 (*n* = 1)

### DNA extraction

Before DNA extraction, each intestinal content sample was transferred (up to 200 mg) to a 2 ml microcentrifuge tube containing 200 mg of magnetic beads. The samples were then vortexed (Voterx-5 Qilinbeier, Scientific Industries, New York, USA) at maximum speed and incubated at 56 °C until the intestinal content samples were completely lysed. Then DNA was extracted using the DNeasy Blood & Tissue Kit (Cat. #69506; Qiagen, Hilden, Germany) according to the manufacturer’s instructions. The extracted DNA was stored at − 20 °C in a freezer until further use.

### PCR amplification

*Enterocytozoon bieneusi* was identified and genotyped using nested PCR, in which an approximately 390 bp nucleotide fragment, including 243 bp of the ITS region, was amplified [29]. Two pairs of primers, EBITS3/EBITS4 and EBITS1/EBITS2, were used in the first and second PCR amplifications, respectively. The two cycling parameters were as follows: 35 cycles at 94 °C for 30 s, 57 °C for 30 s and 72 °C for 40 s, and 30 cycles at 94 °C for 30 s, 55 °C for 30 s and 72 °C for 40 s, with both having a final extension step at 72 °C for 10 min [30]. A positive control (DNA of human-derived genotype D) and a negative control (DNase-free water) were used for the primary and secondary PCR tests to ensure accuracy. TaKaRa *Taq* DNA polymerase (TaKaRa Bio Inc., Tokyo, Japan) was used and the secondary PCR products were visualized using agarose gel electrophoresis and staining the 1.5% gel with GelRed (Biotium Inc., Hayward, CA, USA).

### Nucleotide sequencing and analysis

MEGA 7 (http://www.megasoftware.net) was used to align and compare the nucleotide sequences obtained in the present study with each other and the referenced *E. bieneusi* sequences obtained from GenBank (http://www.ncbi.nlm.nih.gov). If the genotypes obtained in this study were identical to the known genotypes in GenBank, they would be given the first published names. If the ITS sequences had nucleotide deletions, insertions, and substitutions that were different from those of known genotypes, they were considered as novel genotypes. All the genotypes were identified on the basis of the 243 bp of the ITS region of the rRNA gene of *E. bieneusi* according to the established nomenclature system [29].

### Phylogenetic analysis

A Maximum Likelihood tree was constructed for *E. bieneusi* by using MEGA 7 (http://www.megasoftware.net) to evaluate the genetic relationship between the ITS genotypes of *E. bieneusi* obtained in this study and known genotypes deposited in GenBank. The Tamura 3-parameter model was used, and bootstrap analysis with 1000 replicates was performed to assess the reliability of the tree. Nucleotide sequences of *Enterocytozoon hepatopenaei* from shrimp (GenBank: MH260592) and *E. bieneusi* from the red kangaroo (GenBank: KY706128) were used as the outgroup for the phylogenetic analysis.

## Results

### Occurrence of *E. bieneusi*

A total of 498 intestinal contents of Himalayan marmots and Alashan ground squirrels were screened for the presence of *E. bieneusi* by amplifying the ITS region with nested PCR. Fifty samples were successfully amplified and sequenced; the overall infection rate of *E. bieneusi* was 10.0% (50/498), with 11.8% (47/399) for Himalayan marmots and 3.0% (3/99) for Alashan ground squirrels. *Enterocytozoon bieneusi* was detected in all five investigated areas. For Himalayan marmots, the infection rates varied from 7.8% to 17.2% in 4 areas (Table 1).

### Genetic characterizations and distribution of *E. bieneusi* genotypes

Based on sequence analysis of the ITS region, 7 genotypes were identified from the 50 *E. bieneusi* isolates: 1 known genotype, YAK1 (GenBank: MK843246, *n* = 18), and 6 novel genotypes, named as ZY37 (GenBank: MN378367, *n* = 27), HN39 (GenBank: MN378365, *n* = 1), HN96 (GenBank: MN378366, *n* = 1), SN45 (GenBank: MN378369, *n* = 1), XH47 (GenBank: MN378370, *n* = 1) and ZY83 (GenBank: MN378371, *n* = 1). Genotypes ZY37, YAK1, SN45, XH47 and ZY83 were detected in the Himalayan marmots, with genotype ZY37 showing a predominance (57.4%, 27/47). Genotypes HN39, HN96 and YAK1 were detected in the Alashan ground squirrels (Table 1). A total of 14 nucleotide polymorphic sites were observed, and each genotype differed by 1–9 single-nucleotide polymorphisms (Table 2).

**Table 2:** Nucleotide variation in ITS gene region of *E. bieneusi* isolates obtained in this study

Genotype	GenBank ID	Nucleotide at position (ITS)													
		17	30	31	51	52	92	93	113	117	125	158	177	192	219
Novel															
HN39	MN378365	G	T	G	G	T	G	C	C	T	A	T	C	G	G
HN96	MN378366	G	**C**	**A**	G	T	G	**T**	**T**	**G**	A	**C**	**T**	**A**	**A**
SN45	MN378369	G	**C**	**A**	G	T	G	**T**	**T**	**G**	**G**	**C**	C	**A**	**A**
XH47	MN378370	**T**	**C**	**A**	G	T	G	**T**	**T**	**G**	A	**C**	C	**A**	**A**
ZY37	MN378367	G	**C**	**A**	–	–	G	**T**	**T**	**G**	A	**C**	C	**A**	**A**
ZY83	MN378371	G	**C**	**A**	G	T	–	–	**T**	**G**	A	**C**	C	**A**	**A**
Known															
YAK1	MK843246	G	**C**	**A**	G	T	G	**T**	**T**	**G**	A	**C**	C	**A**	**A**

*Note*: –, base deletion; bold text, different base compared to HN39

The novel genotype HN39 had 99.59% similarity with genotype D (syn. CEbC, NCF7, Peru9, PigEBITS9, PtEb VI, SHW1 and WL8), with one base substitution (A to G). The other five novel genotypes (ZY37, HN96, SN45, XH47 and ZY83) showed the highest similarity with genotype YAK1: 99.59% for genotypes HN96 (C to T), SN45 (A to G) and XH47 (G to T), with one base substitution, and 99.18% for genotypes ZY37 (T to -, G to -) and ZY83 (G to -, T to -), with two base deletions.

### Phylogenetic relationship of *E. bieneusi* genotypes

**Fig. 1:**
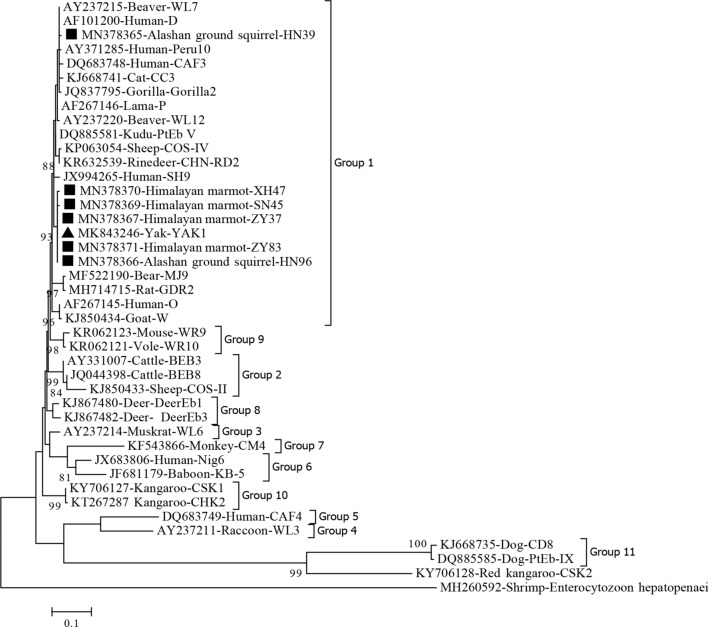
Phylogenetic relationships of *E. bieneusi* genotypes identified in this study and known genotypes published on GenBank inferred using the Maximum-Likelihood method. Genetic distances of the ITS sequences were calculated using the Tamura 3-parameter model. The numbers above the branches are bootstrap values from 1000 replicates. The group terminology for the clusters is based on the Li W et al. [14]. The black triangles and squares indicate the known genotype YAK1 and six novel genotypes identified in the present study, respectively

Phylogenetic analysis of the Maximum Likelihood tree for the ITS sequences of *E. bieneusi* showed that all the genotypes obtained in the present study were classified into group 1. Genotypes ZY37, YAK1, HN96, SN45, XH47 and ZY83 were clustered into one branch, and genotype HN39 was clustered into another branch (Fig. 1).

## Discussion

Rodents, especially wild ones, can act as vectors for numerous pathogens, and some of the pathogens, including *E. bieneusi* are zoonotic. *Enterocytozoon bieneusi* has been reported in some wild rodent species: with a prevalence of 38.9% (121/311) in wild bank voles and wild mice from Poland [25]; 26.8% (38/142) in wild beavers, chipmunks, deer mice, muskrats, porcupines, squirrels, voles, and woodchucks from New York, USA [9]; 10.7% (31/289) in wild mice from Germany/Czech Republic [24]; 10.2% (33/324) in wild beavers and muskrats from Maryland, USA [23]; 7.9% (19/242) in brown rats from Heilongjiang Province, China [19]; 4.0% (8/199) in brown rats and wild mice from Henan Province, China [28]; and 1.1% (3/280) in wild mice from Slovakia [27]. In the present study, *E. bieneusi* was detected in Himalayan marmots (11.8%, 47/399) and Alashan ground squirrels (3.0%, 3/99). The prevalence of *E. bieneusi* is difficult to compare because it may be influenced by many factors, such as the immune status of the hosts, number of samples, experimental methods, and climatic and geographical differences.

**Fig. 2:**
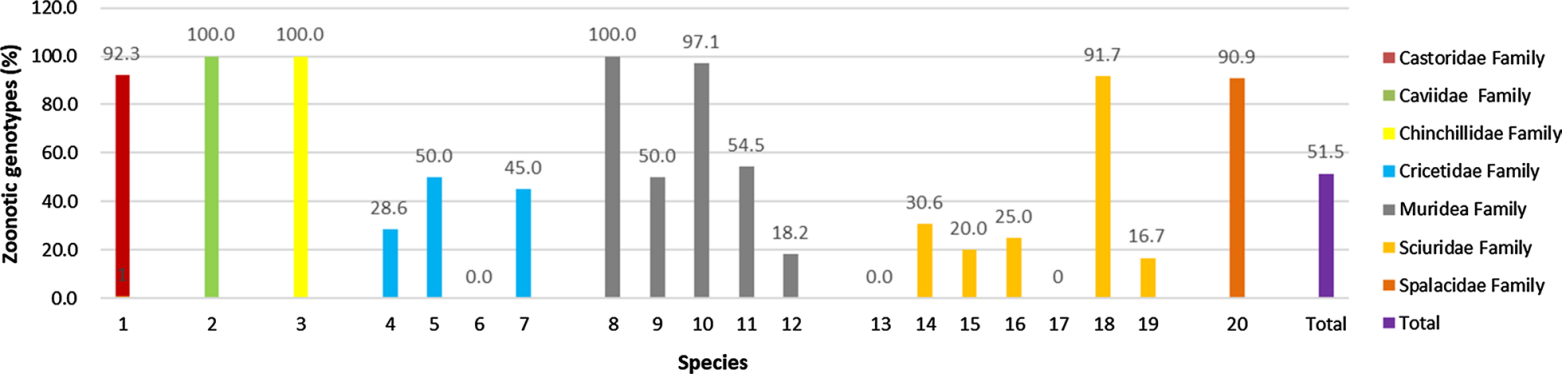
Percentage of zoonotic *E. bieneusi* genotypes in rodents worldwide. 1, beaver (*Castor fiber*); 2, guinea pig (*Cavia porcellus*); 3, chinchilla (*Chinchilla lanigera*); 4, bank vole (*Myodes glareolus*); 5, boreal red-backed vole (*Myodes gapperi*) and meadow vole (*Microtus pennsylvanicus*) (No separate genotypes information provided in the original reference); 6, deer mouse (*Peromyscus* sp.); 7, muskrat (*Ondatra zibethicus*); 8, brown rat (*Rattus norvegicus*); 9, brown rat (*Rattus norvegicus*) and house mouse (*Mus musculus*) (No separate genotypes information provided in the original reference); 10, house mouse (*Mus musculus*); 11, striped field mouse (*Apodemus agrarius*); 12, yellow-necked mouse (*Apodemus flavicollis*); 13, Alashan ground squirrel (*Spermophilus alashanicus*); 14, chipmunk (*Eutamias asiaticus*); 15, eastern chipmunk (*Tamias striatus*); 16, eastern gray squirrel (*Sciurus carolinensis*), red squirrel (*Sciurus vulgaris*) and southern flying squirrel (*Glaucomys volans*) (No separate genotypes information provided in the original reference)); 17, Himalayan marmot (*Marmota himalayana*); 18, red-bellied tree squirrel (*Callosciurus erythraeus*); 19, woodchuck (*Marmota monax*). 20, bamboo rat (*Rhizomys sinensis*). The bar chart was produced from the data presented in Additional file 1: Table S1

Because of the high degree of genetic polymorphisms in the ITS region of the rRNA gene, sequence analysis of the ITS region is regarded as the standard method for identifying and genotyping *E. bieneusi* isolates [29]. In 2002, Buckholt et al. [30] proposed that if enough isolates are sequenced, all 243 nucleotides in the ITS region can be found to be polymorphic. In fact, numerous studies on genotyping of the ITS region of *E. bieneusi* isolates provide further information on the transmission routes and genetic characterization of *E. bieneus*i. In the present study, seven genotypes were identified from 50 *E. bieneusi* isolates, with genotypes ZY37, YAK1, SN45, XH47 and ZY83 in the Himalayan marmots and genotypes YAK1, HN39 and HN96 in the Alashan ground squirrels. To date, 63 genotypes of *E. bieneusi* including those obtained in this study, have been identified in seven rodent families from seven countries: six genotypes (D, EbpC, WL7, WL9, WL12 and WL15) in the family Castoridae; one genotype (Peru16) in the family Caviidae; two genotypes (BEB6 and D) in the family Chinchillidae; 16 genotypes (D, EbpC, Peru11, Type IV, WL4, WL6, WL10, WL14, WL15, WL20, WL21, WL23, WL25, WR2, WR6, WR10) in the family Cricetidae; 22 genotypes (BEB6, C, CD6, CHG2, CZ3, D, EbpA, gorilla 1, H, Peru6, Peru8, Peru16, PigEBITS5, S6, WR1, WR3 to WR9) in the family Muridae; and 27 genotypes (CE01, CHG9, D, EbpC, HN39, HN96, Horse2, Nig7, PtEb V, S7, SC02, SCC-1 to SCC-4, SN45, Type IV, WL4, WL20 to WL23, WW6, XH47, YAK1, ZY37 and ZY83) in the family Sciuridae; 6 genotypes (BR1, BR2, D, EbpA, J, PigEBITS7) in the family Spalacidae. In humans, 34.9% (22/63) of the 63 genotypes have been identified: C, CZ3, D, EbpA, EbpC, H, Peru 6, Peru8, Peru11, Peru16, PigEBITS5, PigEBITS7, S6, SC02, Type IV, WL7, WL12, and WL15 in zoonotic group 1; BEB6 and J in group 2; Nig 7 in group 6; and S7 in group 10 (Additional file 1: Table S1). Zoonotic *E. bieneusi* genotypes have been identified in 12 of 13 epidemiological studies. The prevalence of zoonotic genotypes was observed to vary in different host species ranging between 0–100% (Fig. 2). All the *E. bieneusi* genotypes have been found in humans in some epidemiological studies, including those conducted in chinchillas and brown rats in China [18, 19], guinea pigs in Peru [26], and house mice in Germany/Czech Republic and Slovakia [24, 27] (Additional file 1: Table S1). These results indicate that rodents infected with *E. bieneusi* may play an important role in its transmission to humans and become an important source of water contamination in the areas investigated in this study.

To date, the known genotype YAK1 has been detected in only domestic yaks in QTPA, Qinghai Province, China [31]. Although no zoonotic *E. bieneusi* genotypes were obtained in this study, genotype YAK1 and six novel genotypes were clustered into group 1 in the phylogenetic analysis, indicating the zoonotic ability and public health significance of these genotypes. In fact, the genotype HN39 shows only one base difference when compared with D (pathogenic in humans). In the investigated areas, wild Himalayan marmots and Alashan ground squirrels share the same pasture with humans, herbivorous animals (cattle, sheep and yaks, etc.), and other wild animals (plateau pikas and Tibetan antelopes, etc.). Wild animals usually have strong migration habits and a wide range of activity. Infection occurs through oral ingestion of the environmentally resistant spores of *E. beneusi* from contaminated feces, food, drink and pasture (for grazing animals), which provides feasible opportunities for the transmission of the spores from wild Himalayan marmots and Alashan ground squirrels to humans and animals. Therefore, these animals infected with *E. bieneusi* can be potential sources of microsporidiosis in humans and other animals and pose a threat to public health and ecological security.

Generally, the ITS region is 243-bp long in natural *E. bieneusi* genotypes. However, in the present study, amplicons of 241 bp were observed in the ITS region of the novel genotypes ZY37 and ZY83. In fact, length variations in the ITS region of *E. bieneusi* have been found in at least 15 *E. bieneusi* genotypes. Amplicons of less than 243 bp in the ITS region have been reported for a few genotypes: 241 bp in genotype MAY1 from a renal transplant recipient in France [32]; CHN-RR1 from rex rabbits in China [33]; CHN3, CHN4 and CHN5 from diarrheal children in China [34]; YN249 from a Yao person in China [35]; and PigEb12 and PigEb15 from pigs in Brazil [38] and 242 bp in genotype CAF4 from HIV-positive patients in Gabon and villagers in Cameroon [36]. In contrast, amplicons of more than 243 bp in the ITS region have been found in some genotypes: 244 bp in genotype SCBB1 from captive black bears in China [37], as well as genotypes HNM-V, HNM-VI, and HNM-VII from captive long-tailed macaques in China [15]; 245 bp in genotype PigEb16 from pigs in Brazil [38], and genotype Q from patients with diarrhea in Switzerland [39]. These observations indicate that base deletions and insertions can occur in the ITS region of the rRNA gene of *E. bieneusi*.

## Conclusions

To the best of our knowledge, this is the first report of the identification and genotyping of *E. bieneusi* in wild Himalayan marmots and Alashan ground squirrels in QTPA, Gansu Province, China. All seven genotypes (ZY37, YAK1, HN39, HN96, SN45, XH47 and ZY83) were classified into group 1, suggesting the possibility of zoonotic and cross-species transmission. Further molecular epidemiological surveys are required to assess the importance and transmission dynamics of *E. bieneusi* in other animals and humans, including local residents and tourists in close contact with pasture. Currently, no effective treatment or vaccine is available for microsporidiosis caused by *E. bieneusi*. Therefore, it is necessary to make people aware of the potential risk of zoonotic transmission of this disease and importance of strengthening management practices and surveillance for *E. bieneusi* in wild animals.

## Supplementary information

**Additional file 1: Table S1.** Genotypes of *E. bieneusi* in rodents worldwide.

## Data Availability

The data supporting the conclusions of this article are included within the article and its additional file. Nucleotide sequences were deposited in the GenBank database under the Accession numbers MN378365-MN378367, MN378369-MN378371.

## Abbreviations

QTPA: Qinghai-Tibetan Plateau area
ITS: ribosomal internal transcribed spacer
PCR: polymerase chain reaction
