# Supplementary material for: Identification and genotyping of Enterocytozoon bieneusi in wild Himalayan marmots (Marmota himalayana) and Alashan ground squirrels (Spermophilus alashanicus) in the Qinghai-Tibetan Plateau area (QTPA) of Gansu Province, China
Source: Parasit Vectors. 2020 Jul 22;13:367. doi: 10.1186/s13071-020-04233-9 (PMC7376879; doi:10.1186/s13071-020-04233-9)
Supplement: Supplementary file 1 — Additional file 1: Table S1. Genotypes of E. bieneusi in rodents worldwide. [file 13071_2020_4233_MOESM1_ESM.docx]

**Additional file 1: Table S1. Genotypes of** E**.** bieneusi **in rodents worldwide.**

| Family | Species (Latin name) | Country | Genetic group: Genotypes (n) | % zoonotic genotypes | Ref |
| --- | --- | --- | --- | --- | --- |
| [Castoridae](https://www.ncbi.nlm.nih.gov/Taxonomy/Browser/wwwtax.cgi?mode=Undef&id=29132&lvl=3&keep=1&srchmode=1&unlock) | Beaver (*Castor fiber*) | USA | 1: **EbpC** (5), **D** (4), **WL7**, WL9, **WL12**, **WL15** (1 each) | 92.3 | [23] |
| [Caviidae](https://www.ncbi.nlm.nih.gov/Taxonomy/Browser/wwwtax.cgi?mode=Undef&id=10139&lvl=3&keep=1&srchmode=1&unlock) | Guinea pig (*Cavia porcellus*) | Peru | 1: **Peru16** (10) | 100.0 | [26] |
| [Chinchillidae](https://www.ncbi.nlm.nih.gov/Taxonomy/Browser/wwwtax.cgi?mode=Undef&id=10150&lvl=3&keep=1&srchmode=1&unlock) | Chinchilla (*Chinchilla lanigera*) | China | 1: **D** (2); 2: **BEB6** (3) | 100.0 | [18] |
| [Cricetidae](https://www.ncbi.nlm.nih.gov/Taxonomy/Browser/wwwtax.cgi?mode=Undef&id=337677&lvl=3&keep=1&srchmode=1&unlock) | Bank vole (*Myodes glareolus*) | Poland | 1: **D** (2), WR2 (1); 2: WR6 (2); 9: WR10 (2) | 28.6 | [25] |
|  | Boreal red-backed vole (*Myodes gapperi)*; Meadow vole (*Microtus pennsylvanicus*) | USA | 1: **Peru11** (2), WL21 (2), **Type IV** (1), WL20 (1) | 50.0 | [9] |
|  | Deer mouse (*Peromyscus* sp.) | USA | 3: WL4 (10), WL23 (2), WL25 (1) | - | [9] |
|  | Muskrat (*Ondatra zibethicus*) | USA | 1: **WL15** (4), **EbpC** (3), **D** (2), WL10 (1), WL14 (1); 3: WL4 (8), WL6 (1) | 45.0 | [23] |
| Subtotal |  |  | WL4 (18), **WL15** (4), **D (4), EbpC** (3), **Peru11**, WL21, WL23, WR6, WR10 (2 each), **Type IV**, WL6, WL10, WL14, WL20, WL25, WR2 (1 each) | 30.4 |  |
| [Muridae](https://www.ncbi.nlm.nih.gov/Taxonomy/Browser/wwwtax.cgi?mode=Undef&id=10066&lvl=3&keep=1&srchmode=1&unlock) | Brown rat (*Rattus norvegicus*) | China | 1: **D** (17), **Peru6** (2) | 100.0 | [19] |
|  | Brown rat (*Rattus norvegicus*); House mouse (*Mus musculus*) | China | 1: **D** (2); 2: CD6 (3), **BEB6** (2), CHG2 (1) | 50.0 | [28] |
|  | House mouse (*Mus musculus*) | Germany/Czech | 1: **D** (10), **PigEBITS5** (7), **CZ3** (4), **Peru8** (4), **C** (2), **EbpA** (2), **H** (1), **S6** (1) | 100.0 | [24] |
|  |  | Poland | 1: WR3 (1) | - | [25] |
|  |  | Slovakia | 1: **Peru16** (3) | 100.0 | [27] |
|  | Striped field mouse (*Apodemus agrarius*) | Poland | 1: **D** (6), gorilla 1 (1); 2: WR5 (1); 9: WR8 (2), WR7 (1) | 54.5 | [25] |
|  | Yellow-necked mouse (*Apodemus flavicollis*) | Poland | 1: **D** (2), WR1 (1), WR4 (1); 2: WR6 (6); 9: WR9 (1) | 18.2 | [25] |
| Subtotal |  |  | **D** (37), **PigEBITS5** (7), WR6 (6), **CZ3** (4), **Peru8** (4), CD6 (3), **Peru16** (3), **BEB6**, **C**, **EbpA**, **Peru6**, WR8 (2 each), CHG2, gorilla 1, **H**, **S6**, WR1, WR3 to WR5, WR7, WR9 (1 each) | 77.4 |  |
| [Sciuridae](https://www.ncbi.nlm.nih.gov/Taxonomy/Browser/wwwtax.cgi?mode=Undef&id=55153&lvl=3&keep=1&srchmode=1&unlock) | Alashan ground squirrel (*Spermophilus alashanicus*) | China | 1: HN39, HN96, YAK1 (1 each) | - | This study |
|  | Chipmunk (*Eutamias asiaticus*) | China | 1: **D** (6), CHG9 (2); 6: **Nig7** (4); 10: SCC-1 (17), SCC-2 (9), **S7** (5), SCC-3 (5), SCC-4 (1) | 30.6 | [20] |
|  | Eastern chipmunk (*Tamias striatus*) | USA | 1: **Type IV** (1); 3: WL4 (3), WL23 (1) | 20.0 | [9] |
|  | Eastern gray squirrel (*Sciurus carolinensis*); Red squirrel (*Sciurus vulgaris*); Southern flying squirrel (*Glaucomys volans*) | USA | 1: **Type IV** (3), PtEb V (1), WL21 (1); 3: WL4 (5); 4: WW6 (2) | 25.0 | [9] |
|  | Himalayan marmot (*Marmota himalayana*) | China | 1: ZY37 (27), YAK1 (17), SN45, XH47, ZY83 (1 each) | - | This study |
|  | Red-Bellied Tree Squirrel (*Callosciurus erythraeus*) | China | 1: **D** (18), **EbpC** (3), **SC02** (1); 6: CE01 (1), Horse2 (1) | 91.7 | [17] |
|  | Woodchuck (*Marmota monax*) | USA | 1: **Type IV** (1), WL20 (1); 3: WL4 (2), WL22 (1), WW6 (1) | 16.7 | [9] |
| Subtotal |  |  | ZY37 (27), **D** (24), YAK1 (18), SCC-1 (17), WL4 (10), SCC-2 (9), **S7**, SCC-3, **Type IV** (5 each), **Nig7** (4), **EbpC** (3), WW6 (3), CHG9 (2), CE01, HN39, HN96, Horse2, PtEb V, **SC02**, SCC-4, SN45, WL20 to WL23, XH47, ZY83 (1 each) | 28.8 |  |
| [Spalacidae](https://www.ncbi.nlm.nih.gov/Taxonomy/Browser/wwwtax.cgi?mode=Undef&id=337664&lvl=3&keep=1&srchmode=1&unlock) | Bamboo rat (*Rhizomys sinensis*) | China | 1: **D** (17), **EbpA** (1), **PigEBITS7** (1); 2: BR1, BR2, **J** (1 each) | 90.9 | [21] |
| Total |  |  | **D** (88), WL4 (28), ZY37 (27), YAK1 (18), SCC-1 (17), **Peru16** (13), **EbpC** (11), SCC-2 (9), WR6 (8), **PigEBITS5** (7), **Type IV** (6), **BEB6**, **S7**, SCC-3, **WL15** (5 each), **CZ3**, **Nig7**, **Peru8** (4 each), CD6, **EbpA**, WL21, WL23, WW6 (3 each), **C**, CHG9, **Peru6**, **Peru11**, WL20, WR8, WR10 (2 each), BR1, BR2, CE01, CHG2, gorilla 1, **H**, HN39, HN96, Horse2, **J**, **PigEBITS7**, PtEb V, **S6**, **SC02**, SCC-4, SN45, WL6, **WL7**, WL9, WL10, **WL12**, WL14, WL22, WL25, WR1 to WR5, WR7, WR9, XH47, ZY83 (1 each) | 51.5 |  |

Note: Two E**.** bieneusi genotypes in Table S1 have been changed to the first published names instead of genotype names described in original papers: CHY1 to S7 [20], CHG14 to CD6 [28].

Bold genotypes indicate the genotypes found in humans previously.

The invalid genotype Row has not been mentioned in the table [22].
